# TRPCs: Influential Mediators in Skeletal Muscle

**DOI:** 10.3390/cells9040850

**Published:** 2020-04-01

**Authors:** Jun Hee Choi, Seung Yeon Jeong, Mi Ri Oh, Paul D. Allen, Eun Hui Lee

**Affiliations:** 1 Department of Physiology, College of Medicine, The Catholic University of Korea, Seoul 06591, Korea; 2 Department of Biomedicine & Health Sciences, Graduate School, The Catholic University of Korea, Seoul 06591, Korea; 3 Leeds Institute of Biomedical & Clinical Sciences, St. James’s University Hospital, University of Leeds, Leeds LS97TF, UK

**Keywords:** skeletal muscle, TRPC, Ca^2+^ entry, SOCE, Duchenne muscular dystrophy

## Abstract

Ca^2+^ itself or Ca^2+^-dependent signaling pathways play fundamental roles in various cellular processes from cell growth to death. The most representative example can be found in skeletal muscle cells where a well-timed and adequate supply of Ca^2+^ is required for coordinated Ca^2+^-dependent skeletal muscle functions, such as the interactions of contractile proteins during contraction. Intracellular Ca^2+^ movements between the cytosol and sarcoplasmic reticulum (SR) are strictly regulated to maintain the appropriate Ca^2+^ supply in skeletal muscle cells. Added to intracellular Ca^2+^ movements, the contribution of extracellular Ca^2+^ entry to skeletal muscle functions and its significance have been continuously studied since the early 1990s. Here, studies on the roles of channel proteins that mediate extracellular Ca^2+^ entry into skeletal muscle cells using skeletal myoblasts, myotubes, fibers, tissue, or skeletal muscle-originated cell lines are reviewed with special attention to the proposed functions of transient receptor potential canonical proteins (TRPCs) as store-operated Ca^2+^ entry (SOCE) channels under normal conditions and the potential abnormal properties of TRPCs in muscle diseases such as Duchenne muscular dystrophy (DMD).

## 1. Introduction

Regarding skeletal muscle, intracellular Ca^2+^ from the SR (similar to the ER in the other types of cells) has been thought to be the only Ca^2+^ source for excitation-contraction (EC) coupling during skeletal muscle contraction [1,2,3,4,5,6]. A new study found that the depletion of Ca^2+^ from the SR induces extracellular Ca^2+^ entry (also called Ca^2+^ influx) into mouse skeletal muscle fibers, and this extracellular Ca^2+^ entry is independent of the well-known dihydropyridine receptor (DHPR), an L-type Ca^2+^ channel in the plasma membrane [7]. These findings raise the following questions about extracellular Ca^2+^ entry in skeletal muscle cells: when, how, and why is extracellular Ca^2+^ entry used in skeletal muscle cells?

Before going further, please note that several terms are used to define skeletal muscle cells at different stages or conditions: satellite cells (skeletal muscle stem cells), myoblasts (a proliferative form of satellite cells after losing stemness), myotubes (multinucleated skeletal muscle cells that are differentiated in vitro), and adult skeletal muscle fibers (or simply adult fibers, mature muscle cells isolated from the skeletal muscles of animals or human biopsy). Myoblasts are differentiated into muscle fibers (or myotubes in vitro) during the development or regeneration of skeletal muscle, such as during postnatal growth or regeneration after injury. The former scenario is called differentiation, while the latter scenario is called terminal differentiation.

## 2. Store-Operated Ca^2+^ Entry (SOCE) as a Mechanism of Extracellular Ca^2+^ Entry into Skeletal Muscle

The channel proteins responsible for extracellular Ca^2+^ entry in response to Ca^2+^ depletion from internal Ca^2+^ stores (the ER or SR) in various cells are defined as SOCE channels (these include the Ca^2+^ release-activated channels (CRAC) and capacitative Ca^2+^ entry (CCE) channels defined during the late 1990s and early 2000s) [8,9,10]. The functions of SOCE channels were initially unclear in these studies, although the existence of SOCE channels was previously described in studies on Ca^2+^ movement in skeletal muscle [7,11]. Further studies on SOCE channels using skeletal muscle myotubes or isolated adult skeletal muscle fibers have been successfully performed and have revealed the roles of SOCE in skeletal muscle physiology and pathophysiology [12,13,14,15,16,17,18,19]. SOCE is believed to be important in the refilling of the SR with Ca^2+^ (such as after or during tetanic stimulations or fatigue) and in maintaining the SR Ca^2+^ content at a steady state for maximal skeletal muscle performance [13,15,20,21].

Generally, upon Ca^2+^ depletion from the ER or SR, STIM (stromal interaction molecule, which is an ER Ca^2+^ sensor) self-oligomerizes and activates SOCE by binding to Ca^2+^ entry channels in the plasma membrane (usually Orai1 in many cell types) [22,23,24,25,26,27]. The oligomerized complexes of STIMs and Orai1 that form during SOCE are called puncta. Known variants of STIM and Orai, namely, STIM1, STIM2, STIM1L, Orai1, Orai2, and Orai3, are expressed in different tissues [28]. STIMs have a luminal N-terminus with a Ca^2+^-binding canonical EF-hand and a sterile α-motif, a single transmembrane domain, and a cytosolic C-terminus with three coiled-coil domains (that overlap with the STIM–Orai-activating region/CRAC activation domain (SOAR/CAD)), a Pro/Ser-rich domain, and a Lys-rich domain [28]. Orais are predicted to have four transmembrane domains and share structural similarities with the γ subunit of the L-type Ca^2+^ channel [28,29].

Concerning skeletal muscle cells, STIM-dependent SOCE-mediating channels are divided into two groups: Orai channels (main players) and transient receptor potential canonical channels (TRPCs, emerging players) [8,13,15,30,31,32,33,34,35,36,37,38]. Both groups of SOCE-mediating channels are efficiently and closely located in the triad junction, a specialized macrostructure composed of a parallel transverse (t)-tubule and two opposing SR membranes in skeletal muscle cells [1,2,3,4,5,6]. SOCE through Orai in skeletal muscle has been well studied. Patients with mutations in Orai1 manifest skeletal myopathies and immunodeficiencies [39], for example. Added to the interaction of Orai1 with STIMs, TRPCs also function as SOCE channels in skeletal muscle cells, and the heteromerization of TRPCs with STIM1 allows for this SOCE channel function [35,36,37]. Some TRPCs in skeletal muscle cells also may serve as Ca^2+^ entry channels but not through SOCE mechanisms [38]. The properties of Orai and the TRPCs that are known to be expressed in skeletal muscle cells and their functions are discussed below.

## 3. Orais and STIMs in Skeletal SOCE

Regarding skeletal muscle, the formation of puncta is achieved as a part of the terminal differentiation process from myoblasts to myotubes, which increases the skeletal SOCE kinetics to less than one second by skipping punctum formation (i.e., temporal advantage due to the positioning at the triad junction) [15,40,41,42,43]. Orai1 is the major SOCE-mediating channel in skeletal muscle cells [13,17,19,21,43,44]. Knockout of Orai1 in mice induces defects in muscle development and terminal differentiation and reduces muscle mass due to the absence of SOCE through Orai1 [19,33,45]. Mutations in the Orai1 gene, and subsequently abnormal SOCE, are closely related to skeletal muscle diseases. Patients with an Orai1 mutation (R91W) show muscular hypotonia along with severe combined immunodeficiency (SCID), mainly due to depressed SOCE [25]. Other patients with A103E/L194P Orai1 mutations also show muscle weakness and hypotonia [39]. Patients with constitutively active Orai1 mutants (G98S, V107M, or T184M) show tubular aggregate myopathy associated with enhanced SOCE [46]. A murine animal model of DMD (i.e., mdx mice) shows excessive SOCE due to increased Orai1 expression [47]. SOCE through Orai1 has been shown to participate in the maintenance of Ca^2+^ levels in both the cytosol and SR at rest in mouse skeletal myotubes [48]. Orai1 interacts with Mitsugumin 53 and this interaction has been shown to enhance Ca^2+^ entry through Orai1 via a SOCE mechanism in mouse skeletal myotubes [49]. The roles of Orai2 or Orai3 in skeletal muscle are not well studied. Like Orai1, Orai2 and Orai3 are expressed in C2C12 myoblasts and, whereas Orai1 expression is increased during the terminal differentiation of C2C12 myoblasts, the expression levels of Orai2 and Orai3 are not changed [50]. Additionally, the mRNA for Orai2 and Orai3 is detected at lower levels than that for Orai1 during all stages [50].

STIM1, STIM2, and STIM1L are expressed in skeletal muscle cells, and STIM1 is directly involved in the normal development and functions of skeletal muscle [13,32,42,43,51,52]. STIM1 knockdown inhibits SOCE and SR Ca^2+^ refilling and subsequently impairs the terminal differentiation of myoblasts to myotubes, whereas STIM1 overexpression accelerates terminal differentiation by increasing SOCE [32,53,54]. The conditional knockout of STIM1 in mouse skeletal muscle induces defects in neonatal skeletal muscle growth and differentiation, and the defects induce a reduction in body mass and perinatal lethality [33,55]. Mutations in the STIM1 gene also are associated with SCID and also are accompanied by skeletal muscle atrophy and myopathy due to a reduction in SOCE [33,56]. Myotubes from mdx mouse models show higher expression of STIM1 with changes in other Ca^2+^-handling proteins [57,58]. A muscular hypotonia-associated STIM1 mutation at R429 induces abnormalities in intracellular Ca^2+^ movement, mitochondria and SOCE in mouse skeletal myotubes [59]. Added to STIM1 acting as a SOCE-mediating protein in skeletal muscle, it also mediates the intracellular Ca^2+^ distribution between the SR and the cytosol by regulating sarcoplasmic/endoplasmic reticulum Ca^2+^-ATPase 1a (SERCA1a) activity without affecting SOCE [60].

STIM1L, a splice variant of STIM1 with additional residues in the cytosolic region, has been identified in skeletal muscle [42,43,52]. The silencing of STIM1L induces a significant delay in the activation of SOCE and the formation of small myotubes, and the effects of STIM1L overexpression are the opposite of those caused by STIM1L silencing [34,42,52,61]. Added to the positional advantage of STIMs and Orais at the triad junction (i.e., the preformation of puncta), it has been proposed that the formation of a permanent STIM1L–Orai1 complex also could be responsible for the faster activation of SOCE in skeletal muscle cells than in other types of cells [42].

STIM2, another isoform of STIM1, is expressed in skeletal muscle [51]. The silencing of STIM2 decreases both the type 1 ryanodine receptor (RyR1, an internal Ca^2+^ channel in the SR membrane) activity and SOCE, and induces defects in the terminal differentiation of myoblasts into myotubes [51,62,63]. STIM1 and STIM2 are functionally redundant because the overexpression of either STIM1 or STIM2 alleviates most of the effects of STIM2 or STIM1 silencing on SOCE and differentiation [51]. STIM2 also regulates intracellular Ca^2+^ distribution by attenuating SERCA1a activity in mouse skeletal myotubes [62], which is similar to the regulation of intracellular Ca^2+^ distribution by STIM1, but the mechanisms by which STIM2 and STIM1 regulate SERCA1a activity are different [60].

To compare Orai1-mediated skeletal SOCE and cardiac or smooth muscle SOCE, please refer to a recent brief review article [64].

## 4. General Aspects of TRPCs

The transient receptor potential (TRP) superfamily includes more than 28 related members in mammalian tissues and is ubiquitously expressed in many types of cells [65,66,67,68,69,70,71]. Most TRP channels are non-selective, non-voltage-dependent (or very weak voltage-dependent) Ca^2+^-permeable cation channels and participate in various cellular processes ranging from sensory events to social behaviors.

The discovery of a spontaneously occurring mutation in *Drosophila melanogaster* that resulted in the lack of a TRP protein (that responds to a continuous light stimulus with a transient receptor potential response) initiated the discovery of the TRP family [72]. Based on amino acid sequences, TRP channels are divided into six subfamilies: TRPC (canonical), TRPV (vanilloid), TRPM (melastatin), TRPA1 (ankyrin), TRPP (polycystin) and TRPML (mucolipin) [67,73,74]. TRP channels form both homomeric and heteromeric oligomers, which confers the different characteristics of these channels due to the heterogeneity of subunit composition and a main disadvantage: the difficulty to study physiological characteristics of heteromers. All TRPs have six transmembrane domains, intracellular N- and C-termini, and a P-loop (i.e., pore-forming region) between the fifth and sixth transmembrane regions, which is similar to that of voltage-dependent K^+^ channels, such as K_v_1.2, except that the fourth transmembrane segment is not positively charged. The proteins homologous to TRP in mammalian cells mediate cellular responses to a large variety of extracellular signals, such as ligands (agonists/antagonists), temperature, pH, osmolarity, oxidative stress, etc. Mutations in TRP homologs induce various diseases ranging from neurodegenerative disorders to different types of cancers [71,75].

The TRPC subfamily is the most closely related to the TRP in *Drosophila melanogaster*, and there are seven TRPCs in mammals (TRPC1 to TRPC7) [67,73,74,76,77,78]. However, TRPC2 is not expressed in humans (i.e., a pseudogene). Based on amino acid sequences, TRPC1, TRPC4 and TRPC5 are considered to form one subgroup, and TRPC3, TRPC6 and TRPC7 form another subgroup. All the TRPCs mediate extracellular cation entry into cells. Their N-terminus contains three to four ankyrin repeats and a putative caveolin binding site that are involved in the targeting of TRPCs to the plasma membrane. Ankyrin repeats are involved in the homo- and hetero-oligomerization of TRPCs, but the native compositions of TRPCs are not fully understood. TRPCs contain calmodulin (CaM)-binding sites that play roles in the Ca^2+^-dependent modulation of TRPC activities. Binding of CaM to the C-terminal CaM and IP_3_R-binding site of TRPC (called the CIRB site) induces Ca^2+^-dependent feedback inhibition [79]. Downstream from the CIRB site of TRPC1, TRPC2, TRPC4 and TRPC5, there is another CaM-binding site (called the CCBII site) and the regulatory effects of CaM by binding to the CCBII site depend on the TRPC subtype: for example, CaM binding to this site causes slow inhibition of TRPC1 and while it activates TRPC5 [79,80]. Both the N- and C-termini contain a coiled-coil region that also participates in the oligomerization of TRPCs.

Generally, the gating of TRPCs is relatively slower than that of other ligand-gated ion channels, and TRPCs are not directly gated by major physiological ligands but involve other regulatory proteins [76,81,82,83,84,85]. The activation of TRPCs involves the activation of phospholipase C (PLC), leading to inositol 1,4,5-triphosphate (IP_3_) and diacylglycerol (DAG) production. Several TRPCs (TRPC1, TRPC3, and TRPC6) are directly coupled to IP_3_ receptors in the ER or are directly regulated by DAG. Regarding details on the modulators of TRPCs, please refer to other well-written review articles [76,81,86,87]. A macroscopic but useful classification to overview TRPC modulators is as follows: (1) CaM kinases, tyrosine kinases (or agonists of tyrosine kinase receptors), and redox (such as H_2_O_2_) are common stimulators of TRPCs; (2) serine-threonine kinases, cyclic AMP (and PKA), and cGMP (and PKG) are common negative modulators of TRPCs; and (3) PKC modulates TRPCs. PKC mostly inhibits TRPCs, but it is an activator when TRPCs are heteromerized with TRPC1. TRPCs are also activated by combined signaling pathways such as phosphatidylinositide 3-kinase, the Rho GTPase Rac1, or phosphatidyl-inositol 4-phosphate 5-kinase [88,89,90]. Several TRPCs are constitutively active without an agonist under some conditions [90,91,92]. As expected, some TRPCs are activated by Ca^2+^ depletion from the ER or SR (i.e., function as SOCE channels) [93,94,95,96,97]. Although this specific property has not been directly studied in skeletal muscle, it has been shown that some TRPCs are mechanosensitive and activated by membrane stretching [38,98,99,100,101,102,103,104,105,106,107]. However, one study suggests that neither TRPC1 or TRPC6 responds to stretch when they are expressed in cells which normally do not express them and another study has suggested the possibility that TRPCs are activated in response to a stimulus from other receptors (in this case G-proteins) which are the actual transducers of stretch [108,109].

## 5. TRPCs as SOCE Channels in Skeletal Muscle

**Figure 1:**
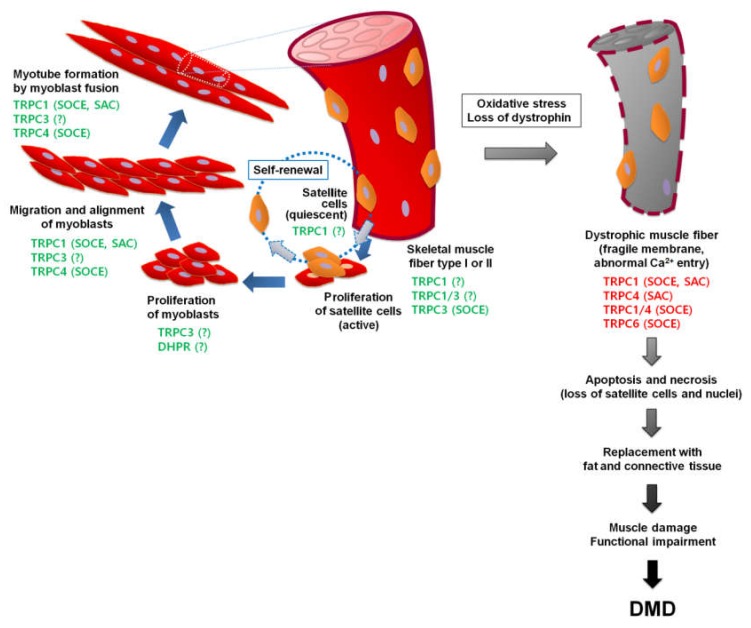
Roles of TRPCs in skeletal muscle. Differentiation or terminal differentiation process is represented by blue arrows. The dashed circle with dashed arrows represents the self-renewal process of satellite cells. Quiescent and active satellite cells are shown in orange and red, respectively. Nuclei are presented as light purple ovals. Green or red TRPCs indicate the involvement of the TRPCs in physiological or pathophysiological processes, respectively. ‘SOCE’ or ‘SAC’ indicates the involvement of TRPCs as a SOCE channel or SAC, respectively. Question mark (?) indicates that the involvement of the channel is established but the working mechanism of the channel is not investigated. SOCE: store-operated Ca^2+^ entry; SAC: stretch-activated Ca^2+^ channel; TRPC: transient receptor potential canonical protein; DMD: Duchenne muscular dystrophy

Various TRP isoforms have been identified in mouse skeletal muscle. Regarding the case of the TRPC subfamily, TRPC1, TRPC3, TRPC4 and TRPC6 expression has mainly been found in skeletal muscle (TRPC2 is a pseudogene in humans) [38,53,61,74,110,111,112]. There are controversies about the expression level of TRPC5 and TRPC7 in skeletal muscle. Using real-time RT-PCR, one study shows no detectable expression of TRPC5 and TRPC7 in mouse tibialis anterior (TA) muscle [38]. However, another study suggests that TRPC5 and TRPC7 expression is higher than those of other subtypes of TRPCs in TA, extensor digitorum longus (EDL) and soleus muscles [113] and in a third study it is found that TRPC5 expression is the same as TRPC1 and TRPC3 in TA muscle but that the expression of TRPC7 is lower [110]. Overall, it seems that the expression level of TRPC5 and TRPC7 is lower than those of other TRPC subtypes in skeletal muscle, suggesting that studies on the expression and function of TRPC5 and TRPC7 in skeletal muscle are needed to resolve this controversy. TRPCs are known to participate in various physiological and pathophysiological events in skeletal muscle, as briefly summarized in Figure 1. Concerning a comparison of TRPC-mediated SOCE or Ca^2+^-entry in skeletal, cardiac or smooth muscle SOCE, please refer to recent review articles [64,76,114,115].

### 5.1. TRPC1 and TRPC4 in Skeletal Muscle

During the development or regeneration of skeletal muscle, the differentiation or terminal differentiation of myoblasts into muscle fibers (or myotubes in vitro) is a critical event [116,117,118]. During the differentiation processes, myoblast migration, alignment, and fusion occur, and Ca^2+^ is a key regulator of the differentiation processes [117,118]. TRPC1 and TRPC4 are important players in the Ca^2+^ supply for the differentiation processes. The expression of TRPC1 is detected in the myoblast stage of C2C12 cells and TRPC1, presumably acting as a stretch-activated mechanosensitive Ca^2+^ channel, participates in the terminal differentiation of C2C12 myoblasts by increasing its expression during the terminal differentiation, followed by a decrease in its expression in mature C2C12 myotubes [107]. Concerning C2C12 myoblasts, TRPC1, which functions as a SOCE channel, also participates in the migration and fusion of myoblasts via calpain activation during the terminal differentiation [119]. Overexpression of TRPC1 in C2C12 myoblasts decreases the nuclear expression of the transcription factor nuclear factor of activated T cells (NFAT) via their function as SOCE channels and this induces negative effects on the terminal differentiation by causing a delay in the onset of terminal differentiation and a formation of thinner myotubes [120].

Muscles from TRPC1-null mice display small fibers (smaller cross-sectional area), less force, and less myofibrillar proteins than muscles from control mice [113]. Found in a heterologous expression system, TRPC1 physically interacts with the a-isoform of the inhibitor of the myogenic family (I-mfa, an inhibitor of basic helix-loop-helix transcription factors such as MyoD and myogenin) [121], which suggests the possibility that TRPC1 participates in the terminal differentiation by interacting with I-mfa. TRPC1 also participates in an adaptive response of skeletal muscle (i.e., muscle regeneration). Down-regulation of TRPC1 expression is required for the regrowth of the mouse soleus muscle after muscle atrophy and the inhibition of calcineurin (CaN) signaling is involved in the down-regulation of TRPC1 expression [122,123]. Additionally, the PI_3_K/Akt/p70S6K pathway plays an important role in muscle regeneration and development and Ca^2+^ entry through TRPC1 has been shown to play a role in the activation of the PI_3_K/Akt/p70S6K pathway during the regenerating of TA and EDL muscles [124]. TRPC1 is expressed in satellite cells and fibroblast growth factor 2 has been shown to trigger the elevation of intracellular Ca^2+^ by activating TRPC1, which participates in the maintenance of the physiological niche of satellite cells on the surface of isolated adult muscle fibers from a mouse by increasing the expression of MyoD [125]. It has been reported that exercise-induced activation of satellite cells in the human vastus lateralis muscle is mediated by cation entry through TRPC1 and the subsequent activation of hepatocyte growth factor [126]. However, the mechanisms activating TRPC1 in satellite cells or during skeletal muscle regeneration have not been addressed. TRPC1 interacts with both TRPC3 and RyR1, and this pair can form heteromeric channels in mouse skeletal myotubes [127]. However, the functional relevance of the heteromeric channels with TRPC1 has not been addressed.

The silencing or dominant-negative suppression of TRPC4, or both TRPC1 and TRPC4 in mouse skeletal muscle fibers or human myotubes, reduces SOCE [38,53], and the overexpression of both TRPC1 and TRPC4 enhances SOCE [128]. TRPC1 and TRPC4 participate in SOCE, which is necessary for the expression of myocyte enhancer factor-2 (MEF2, synergizing the effects of MyoD) and the fusion of human myoblasts to myotubes during terminal differentiation [53].

### 5.2. TRPC3 and TRPC6 in Skeletal Muscle

TRPC3 is another important mediator of the Ca^2+^ supply in the differentiation processes of skeletal muscle and Ca^2+^ entry is a crucial step in the beginning of the skeletal muscle differentiation processes. The expression of TRPC3 is detected in mouse skeletal myoblasts and is sharply upregulated in the early stage of the terminal differentiation (i.e., an initial peak) followed by a gradual decrease during further terminal differentiation to the level of that in myoblasts [127]. Concerning a model where TRPC3 is knocked down with siRNA in α1DHPR-null mouse skeletal myoblasts, there are severe defects in the proliferation of myoblasts and apoptosis during the terminal differentiation, suggesting that the coordinated Ca^2+^ entry through both TRPC3 and DHPR is important for the terminal differentiation process [129]. Interestingly, like TRPC1, TRPC3 expression is down-regulated during the early phase of regrowth of mouse hind limb muscle after atrophy [123]. TRPC3 has been shown to form heteromers with TRPC1 via its ankyrin repeats and regulates the resting cytosolic Ca^2+^ levels in mouse skeletal myotubes [130]. However, it is not understood whether TRPC3 in terminal differentiation and heteromeric TRPC1/3 are acting as SOCE channels. Ca^2+^ entry through TRPC3 via the SOCE mechanism in C2C12 myotubes activates the signaling pathway of CaN/NFAT that determines the myotube phenotype and subsequently alters gene expression from a fast-glycolytic to a slow-oxidative phenotype in a CaN-dependent manner [131]. This phenotype change seems to involve the down-regulation of α-actinin-3 expression, which triggers to increase the expression of proteins that are involved in oxidative metabolism through activation of CaN signaling (such as α-actinin-2, CaN 1.4, acetyl-CoA carboxylase, AMP-activated protein kinase, ATP synthase, succinate-Q oxidoreductase, cytochrome-c oxidoreductase and cytochrome c oxidase) [132]. Regarding an overexpression system, it has been found that Orai1 binds to the N- and C-termini of TRPC3 or TRPC6 and confers SOCE activity to TRPC3 or TRPC6 [35]. However, it is not clear whether TRPC3 or TRPC6 alone act as SOCE channels in human skeletal muscle.

Ca^2+^ entry through TRPC3 via other activation mechanisms has been reported. TRPC3 colocalizes with insulin-sensitive glucose transporter 4 in the t-tubule membrane, and Ca^2+^ entry through DAG-activated TRPC3 enhances insulin-mediated glucose transport [133]. Apart from the role of TRPC3 as a SOCE channel, TRPC3 in mouse skeletal myotubes is required for full EC coupling [134], and the indirect interaction of TRPC3 with RyR1 via other proteins such as, possibly, TRPC1, junctophilin 2, mitsugumin 29, homer 1 and calreticulin has been suggested [127,135]. Thus, it seems that TRPC3 is multifunctional in skeletal muscle.

The role of TRPC6 in skeletal muscle has not been investigated yet, although its expression has been firmly established. Aspects of TRPC6 identified in the non-skeletal muscle cells or heterologous expression systems below help us to predict the roles of TRPC6 in skeletal muscle: TRPC6 forms homo- or heterotetramers with TRPC3 and TRPC7 [136]. TRPC6 also interacts with a number of adaptor proteins, including cytoskeletal proteins [136]. TRPC6 has been shown to be a mechanosensitive channel in several types of cells [137,138]. Mutations in the TRPC6 gene are linked to human diseases. Mutations in the TRPC6 gene (R895C, E897K, or P112Q, gain-of-function mutants) cause familial focal segmental glomerulosclerosis [139,140]. A single nucleotide polymorphism in the TRPC6 gene (C254G) has been linked to idiopathic pulmonary hypertension [141].

Interestingly, STIM1L also interacts with TRPC1, TRPC3, TRPC4, and TRPC6 in skeletal muscle [34,52]. TRPC1 and TRPC4 act as SOCE channels by interacting with STIM1L to promote myogenesis and maintain a fast repetitive Ca^2+^ release in human skeletal myotubes [34], but it is not certain that the same mechanism occurs with TRPC3 and TRPC6.

## 6. TRPCs in Muscular Dystrophy

Human muscular dystrophy is a group of diseases that cause the progressive degeneration of skeletal muscle with severe pain, disability, and finally death [142]. The most severe muscular dystrophy is caused by mutations in dystrophin-associated-proteins (DAP, also called dystrophin–glycoprotein complex (DGC)) on the plasma membrane, although various mutations in different proteins cause human muscular dystrophies [143,144,145]. Dystrophin (427 kDa, an X-chromosome gene, a cytoskeletal protein associated with the plasma membrane) is a scaffold protein for DAP, and syntrophin is the adaptor protein that allows DAP to anchor to various signaling molecules near the plasma membrane, such as ion channels.

### 6.1. Duchenne Muscular Dystrophy (DMD)

DMD is the most prevalent muscular dystrophy (one in 3500 male births) due to a genetic mutation that leads to the complete or partial deficiency of dystrophin and, ultimately after several degeneration/regeneration cycles, leads to the subsequent replacement of skeletal muscle fibers by fat and connective tissue [98,146,147,148,149,150,151,152,153]. First, the lack or partial loss of dystrophin induces a reduction in the expression of DAP and deteriorates the physical link between the cytoskeleton and the extracellular matrix, which makes the plasma membrane more fragile and likely to be torn during stretching stress, such as strong skeletal muscle contraction (concerning more details on stretch-induced muscle damage, please refer to a recent review article on muscle damage [154]). Second, tears in the plasma membrane induce the aggregation of ion channels in the plasma membranes, leading to abnormal channel functions, which disturb Ca^2+^ homeostasis, especially an increase in intracellular Ca^2+^ levels in DMD. The total amount of Ca^2+^ in muscle biopsies from DMD patients was greater than that in muscle biopsies from controls [155]. The Ca^2+^ level in both the SR and cytosol was also higher in mdx myotubes than in control myotubes [156,157,158,159,160]. These increased Ca^2+^ levels activate the aberrant activation of signaling pathways, leading to the loss of satellite cells and nuclei from muscle fibers (subsequent apoptosis and necrosis of muscle fibers) and finally muscle weakness and wasting, impaired muscle regeneration and DMD etiology [161,162,163], as depicted in Figure 1.

Over the past two decades, the dystrophin-deficient mdx mouse, a mouse model of DMD with a relatively milder phenotype than patients with DMD, has been widely used to investigate the mechanism that causes dystrophic muscle damage and degeneration [159,164,165]. Dystrophic myotubes from mdx mice or DMD patients show aberrant Ca^2+^-sensitive signaling pathways due to their high cytosolic Ca^2+^ level: activation of calpains, phospholipase A2, and src kinase, production of reactive oxygen species (ROS), and mitochondrial dysfunction [150,159,166,167,168,169,170,171,172,173,174]. An increase in Ca^2+^ leakage through certain channels was suggested to be a reason for the high cytosolic Ca^2+^ level in dystrophic myotubes (originally called Ca^2+^ leak) [98,157,175,176,177,178,179]. The channels that are responsible for the Ca^2+^ leak are voltage-insensitive, only moderately selective for Ca^2+^, mechanosensitive, have a larger conductance and a higher open probability than those found in wild-type myotubes and are decisively intracellular Ca^2+^ store-dependent (i.e., SOCE) [12,38,180]. Added to the large SOCE, excess mitochondrial Ca^2+^ uptake that induces the functional and structural defects in mitochondria is found in dystrophin-deficient Sol8 myotubes and the expression of mini-dystrophin alleviates the high mitochondrial Ca^2+^ uptake and large SOCE [169]. Biopsies from DMD patients also show ultrastructural abnormalities in mitochondria (without a change in the content of mitochondria) and 60% of maximal respiration activities of control [181,182]. Concerning more details on the role of mitochondria in the regulation of Ca^2+^ signaling in both normal conditions and muscle diseases, refer to a review article [183]. Conversely, studies on muscle-specific transgenic mice overexpressing TRPC3 have indicated that increased Ca^2+^ entry through TRPC3 is sufficient to induce a dystrophic muscle phenotype independent of dystrophin-related membrane tears [184]. These studies suggest that TRPC could be responsible for the abnormal Ca^2+^ entry that induces high cytosolic Ca^2+^ levels in dystrophic myotubes, as summarized in Figure 1.

### 6.2. TRPCs in DMD

The extracellular Ca^2+^ entry in mdx myotubes is approximately twice as high as that in control myotubes [100]. TRPC1 forms a mechanosensitive Ca^2+^ channel (also called a stretch-activated Ca^2+^ channel (SAC) in other articles) in both normal and mdx muscle fibers [38]. The activity of TRPC1 is higher in dystrophic myotubes from mdx mice and human DMD patients, and TRPC1 is responsible for the increased Ca^2+^ entry in mdx myotubes compared with control myotubes [38,98,99,100,101]. Under oxidative stress, NADPH oxidase is a major source of ROS, and its enhanced activity increases the activity of src kinase, the expression of TRPC1 and, subsequently, the mechanosensitive entry of Ca^2+^ through TRPC1 (rather than SOCE through TRPC1), which is believed to be a key mechanism for muscle damage and functional impairment during the pathogenesis of DMD [38,185]. TRPC1 exists as a macromolecular complex anchored to cytoskeletal proteins, such as dystrophin or caveolin-3, in skeletal myotubes or tissues [170,186,187]. Seen in mouse skeletal myotubes, targeting TRPC1 to the plasma membrane requires the binding of TRPC1 to caveolin-3, which also contributes to the higher activity of TRPC1 in mdx myotubes as an SAC [170]. As a cause of higher Ca^2+^ entry in mdx myotubes, higher SOCE is also reported, and TRPC1, as a SOCE channel, is responsible for the higher SOCE [169,186,188].

Caveolin-3 is another protein involved in the pathogenesis of DMD. The expression of caveolin-3, TRPC1 and src kinase, which bind one another, is increased in mdx muscle tissue and shows an irregular membrane distribution [170,189,190,191]. Either the upregulation of caveolin-3 or the suppression of caveolin-3 via genetic ablation deteriorates muscular dystrophies such as DMD or limb-girdle muscular dystrophy-1C [192]. Similar to TRPC1, caveolin-3 also binds to the DAP complex [190,192,193,194]. Therefore, it seems that caveolin-3 is a partner of TRPC1 in the pathogenesis of DMD. Homer proteins are expressed during the terminal differentiation and activate muscle-specific Ca^2+^-dependent gene expression [195]. TRPC1 binds to Homer 1, and mice lacking Homer 1 exhibit myopathy characterized by decreases in muscle fiber cross-sectional area and force generation due to constitutive Ca^2+^ entry thorough TRPC1 (as an SAC) [196].

TRCP4, as an SAC, also contributes to abnormally increased Ca^2+^ entry in adult skeletal muscle fibers from mdx mice [38]. The TRPC4 in mouse skeletal myotubes heteromerizes with TRPC1 and the TRPC1/4 functions as a SOCE channel, the increased Ca^2+^ entry in α1-syntrophin-deficient myotubes is decreased by the repression of either TRPC1 or TRPC4 [128]. TRPC6, as a SOCE channel, also is related to muscular dystrophy. The overexpression of a dominant negative mutant of TRPC6 in mdx or sarcoglycan-deficient mouse models of muscular dystrophy mitigates the dystrophic phenotype by inhibiting SOCE through TRPC6 [184].

Therefore, TRPCs represent valuable therapeutic targets in the treatment of skeletal muscle dystrophies, especially DMD. However, despite extensive research on the mechanisms underlying DMD pathogenesis, no effective treatment is available for patients with DMD. Several clinical trials have attempted to treat DMD patients; however, they were not successful. Aminoglycosides improve the translation of dystrophin in cultured cells; however, an initial trial of gentamicin (one of the aminoglycosides) in DMD patients induces long-term toxic effects with little or no therapeutic benefit [197,198]. Like gentamicin, an initial trial of ataluren (also called PTC124) in DMD patients induces no therapeutic benefit [199]. The high cytosolic Ca^2+^ level at rest in dystrophin-deficient Sol8 myotubes due to higher SOCE is restored to a normal level with the expression of a functional mini-dystrophin or α1-syntrophin, however, unfortunately, the expression of dystrophin in DMD patients is antigenic [169,186,200,201].

## 7. Concluding Remarks

Based on evidence from the studies of many independent laboratories, it seems that SR Ca^2+^ plays several primary roles in skeletal muscle functions, and extracellular Ca^2+^ entry via SOCE and SAC mechanisms (i.e., through Orais or TRPCs) modulates the primary regulatory roles of Ca^2+^ and plays important roles in reinstating skeletal muscle cells to a normal state to be ready for the next cycle of functions.

Until now, many studies on the characterization of TRPCs have been performed using heterologously overexpressed TRPCs, which could be forced to form abnormal multimeric complexes of TRPCs and/or to mislocate within cells. These channels could behave differently from the TRPCs in bona fide cells where TRPCs are normally expressed. Indeed, there are several discrepancies in the activation mechanism of TRPCs and their responses to agonists or antagonists. It seems possible that different genes from different species, different endogenous regulators in exogenous expression systems, and/or the abnormal overexpression of homo- and heteromeric channels could be the reason for the discrepancies. However, it is worth using heterologously and/or overexpressed TRPCs in studying the channel properties and in understanding the regulation mechanisms of TRPCs if it is paralleled by comparative studies using bona fide TRPC-expressing cells and/or using in vivo models of vertebrates and mammals, which could finally dissect the role of TRPCs linked to Ca^2+^ signaling. Therefore, additional extensive studies on the rising star ‘TRPCs’ using bona fide skeletal muscle cells are clearly necessary to help clarify the following questions: when, how, and why is extracellular Ca^2+^ entry used in skeletal muscle cells, and how do STIMs select TRPCs as a functional partner for SOCE rather than Orai1?

## References

[1] Zucchi R., Ronca-Testoni S. (1997). The sarcoplasmic reticulum Ca2+ channel/ryanodine receptor: Modulation by endogenous effectors, drugs and disease states. Pharmacol. Rev..

[2] Cho C.H., Lee K.J., Lee E.H. (2018). With the greatest care, stromal interaction molecule (STIM) proteins verify what skeletal muscle is doing. BMB Rep..

[3] Cho C.H., Woo J.S., Perez C.F., Lee E.H. (2017). A focus on extracellular Ca(2+) entry into skeletal muscle. Exp. Mol. Med..

[4] Lee E.H. (2010). Ca2+ channels and skeletal muscle diseases. Prog. Biophys Mol. Biol..

[5] Lee E.H., Kim D.H., Allen P.D. (2006). Interplay between intra- and extracellular calcium ions. Mol. Cells.

[6] Rios E., Pizarro G., Stefani E. (1992). Charge movement and the nature of signal transduction in skeletal muscle excitation-contraction coupling. Annu. Rev. Physiol..

[7] Kurebayashi N., Ogawa Y. (2001). Depletion of Ca2+ in the sarcoplasmic reticulum stimulates Ca2+ entry into mouse skeletal muscle fibres. J. Physiol..

[8] Prakriya M., Feske S., Gwack Y., Srikanth S., Rao A., Hogan P.G. (2006). Orai1 is an essential pore subunit of the CRAC channel. Nature.

[9] Yeromin A.V., Zhang S.L., Jiang W., Yu Y., Safrina O., Cahalan M.D. (2006). Molecular identification of the CRAC channel by altered ion selectivity in a mutant of Orai. Nature.

[10] Vig M., Beck A., Billingsley J.M., Lis A., Parvez S., Peinelt C., Koomoa D.L., Soboloff J., Gill D.L., Fleig A. (2006). CRACM1 multimers form the ion-selective pore of the CRAC channel. Curr. Biol..

[11] Launikonis B.S., Barnes M., Stephenson D.G. (2003). Identification of the coupling between skeletal muscle store-operated Ca2+ entry and the inositol trisphosphate receptor. Proc. Natl. Acad. Sci. USA.

[12] Ducret T., Vandebrouck C., Cao M.L., Lebacq J., Gailly P. (2006). Functional role of store-operated and stretch-activated channels in murine adult skeletal muscle fibres. J. Physiol..

[13] Stiber J., Hawkins A., Zhang Z.S., Wang S., Burch J., Graham V., Ward C.C., Seth M., Finch E., Malouf N. (2008). STIM1 signalling controls store-operated calcium entry required for development and contractile function in skeletal muscle. Nat. Cell Biol..

[14] Yarotskyy V., Dirksen R.T. (2012). Temperature and RyR1 regulate the activation rate of store-operated Ca(2)+ entry current in myotubes. Biophys. J..

[15] Pan Z., Brotto M., Ma J. (2014). Store-operated Ca2+ entry in muscle physiology and diseases. BMB Rep..

[16] Pan Z., Yang D., Nagaraj R.Y., Nosek T.A., Nishi M., Takeshima H., Cheng H., Ma J. (2002). Dysfunction of store-operated calcium channel in muscle cells lacking mg29. Nat. Cell Biol..

[17] Dirksen R.T. (2009). Checking your SOCCs and feet: The molecular mechanisms of Ca2+ entry in skeletal muscle. J. Physiol..

[18] Stiber J.A., Rosenberg P.B. (2011). The role of store-operated calcium influx in skeletal muscle signaling. Cell Calcium.

[19] Wei-Lapierre L., Carrell E.M., Boncompagni S., Protasi F., Dirksen R.T. (2013). Orai1-dependent calcium entry promotes skeletal muscle growth and limits fatigue. Nat. Commun..

[20] Allen D.G., Lamb G.D., Westerblad H. (2008). Skeletal muscle fatigue: Cellular mechanisms. Physiol. Rev..

[21] Sztretye M., Geyer N., Vincze J., Al-Gaadi D., Olah T., Szentesi P., Kis G., Antal M., Balatoni I., Csernoch L. (2017). SOCE Is Important for Maintaining Sarcoplasmic Calcium Content and Release in Skeletal Muscle Fibers. Biophys. J..

[22] Roos J., DiGregorio P.J., Yeromin A.V., Ohlsen K., Lioudyno M., Zhang S., Safrina O., Kozak J.A., Wagner S.L., Cahalan M.D. (2005). STIM1, an essential and conserved component of store-operated Ca2+ channel function. J. Cell Biol..

[23] Liou J., Kim M.L., Heo W.D., Jones J.T., Myers J.W., Ferrell J.E., Meyer T. (2005). STIM is a Ca2+ sensor essential for Ca2+-store-depletion-triggered Ca2+ influx. Curr. Biol..

[24] Liou J., Fivaz M., Inoue T., Meyer T. (2007). Live-cell imaging reveals sequential oligomerization and local plasma membrane targeting of stromal interaction molecule 1 after Ca2+ store depletion. Proc. Natl. Acad. Sci. USA.

[25] Feske S., Gwack Y., Prakriya M., Srikanth S., Puppel S.H., Tanasa B., Hogan P.G., Lewis R.S., Daly M., Rao A. (2006). A mutation in Orai1 causes immune deficiency by abrogating CRAC channel function. Nature.

[26] Zhang S.L., Yeromin A.V., Zhang X.H., Yu Y., Safrina O., Penna A., Roos J., Stauderman K.A., Cahalan M.D. (2006). Genome-wide RNAi screen of Ca(2+) influx identifies genes that regulate Ca(2+) release-activated Ca(2+) channel activity. Proc. Natl. Acad. Sci. USA.

[27] Park C.Y., Hoover P.J., Mullins F.M., Bachhawat P., Covington E.D., Raunser S., Walz T., Garcia K.C., Dolmetsch R.E., Lewis R.S. (2009). STIM1 clusters and activates CRAC channels via direct binding of a cytosolic domain to Orai1. Cell.

[28] Rosado J.A., Diez R., Smani T., Jardin I. (2015). STIM and Orai1 Variants in Store-Operated Calcium Entry. Front. Pharmacol..

[29] Wissenbach U., Philipp S.E., Gross S.A., Cavalie A., Flockerzi V. (2007). Primary structure, chromosomal localization and expression in immune cells of the murine ORAI and STIM genes. Cell Calcium.

[30] Huang G.N., Zeng W., Kim J.Y., Yuan J.P., Han L., Muallem S., Worley P.F. (2006). STIM1 carboxyl-terminus activates native SOC, I(crac) and TRPC1 channels. Nat. Cell Biol..

[31] Peinelt C., Vig M., Koomoa D.L., Beck A., Nadler M.J., Koblan-Huberson M., Lis A., Fleig A., Penner R., Kinet J.P. (2006). Amplification of CRAC current by STIM1 and CRACM1 (Orai1). Nat. Cell Biol..

[32] Lyfenko A.D., Dirksen R.T. (2008). Differential dependence of store-operated and excitation-coupled Ca2+ entry in skeletal muscle on STIM1 and Orai1. J. Physiol..

[33] Kiviluoto S., Decuypere J.P., De Smedt H., Missiaen L., Parys J.B., Bultynck G. (2011). STIM1 as a key regulator for Ca2+ homeostasis in skeletal-muscle development and function. Skelet Muscle.

[34] Antigny F., Sabourin J., Sauc S., Bernheim L., Koenig S., Frieden M. (2017). TRPC1 and TRPC4 channels functionally interact with STIM1L to promote myogenesis and maintain fast repetitive Ca(2+) release in human myotubes. Biochim. Biophys. Acta Mol. Cell Res..

[35] Liao Y., Erxleben C., Yildirim E., Abramowitz J., Armstrong D.L., Birnbaumer L. (2007). Orai proteins interact with TRPC channels and confer responsiveness to store depletion. Proc. Natl. Acad. Sci. USA.

[36] Lopez J.J., Salido G.M., Pariente J.A., Rosado J.A. (2006). Interaction of STIM1 with endogenously expressed human canonical TRP1 upon depletion of intracellular Ca2+ stores. J. Biol. Chem..

[37] Yuan J.P., Zeng W., Huang G.N., Worley P.F., Muallem S. (2007). STIM1 heteromultimerizes TRPC channels to determine their function as store-operated channels. Nat. Cell Biol..

[38] Vandebrouck C., Martin D., Colson-Van Schoor M., Debaix H., Gailly P. (2002). Involvement of TRPC in the abnormal calcium influx observed in dystrophic (mdx) mouse skeletal muscle fibers. J. Cell Biol..

[39] McCarl C.A., Picard C., Khalil S., Kawasaki T., Rother J., Papolos A., Kutok J., Hivroz C., Ledeist F., Plogmann K. (2009). ORAI1 deficiency and lack of store-operated Ca2+ entry cause immunodeficiency, myopathy, and ectodermal dysplasia. J. Allergy Clin. Immunol..

[40] Launikonis B.S., Stephenson D.G., Friedrich O. (2009). Rapid Ca2+ flux through the transverse tubular membrane, activated by individual action potentials in mammalian skeletal muscle. J. Physiol..

[41] Edwards J.N., Murphy R.M., Cully T.R., von Wegner F., Friedrich O., Launikonis B.S. (2010). Ultra-rapid activation and deactivation of store-operated Ca(2+) entry in skeletal muscle. Cell Calcium.

[42] Darbellay B., Arnaudeau S., Bader C.R., Konig S., Bernheim L. (2011). STIM1L is a new actin-binding splice variant involved in fast repetitive Ca2+ release. J. Cell Biol..

[43] Lee K.J., Woo J.S., Hwang J.H., Hyun C., Cho C.H., Kim D.H., Lee E.H. (2013). STIM1 negatively regulates Ca(2)(+) release from the sarcoplasmic reticulum in skeletal myotubes. Biochem. J..

[44] Roberts-Thomson S.J., Peters A.A., Grice D.M., Monteith G.R. (2010). ORAI-mediated calcium entry: Mechanism and roles, diseases and pharmacology. Pharmacol. Ther..

[45] Carrell E.M., Coppola A.R., McBride H.J., Dirksen R.T. (2016). Orai1 enhances muscle endurance by promoting fatigue-resistant type I fiber content but not through acute store-operated Ca2+ entry. FASEB J..

[46] Bohm J., Bulla M., Urquhart J.E., Malfatti E., Williams S.G., O’Sullivan J., Szlauer A., Koch C., Baranello G., Mora M. (2017). ORAI1 Mutations with Distinct Channel Gating Defects in Tubular Aggregate Myopathy. Hum. Mutat..

[47] Zhao X., Moloughney J.G., Zhang S., Komazaki S., Weisleder N. (2012). Orai1 mediates exacerbated Ca(2+) entry in dystrophic skeletal muscle. PLoS ONE.

[48] Li H., Ding X., Lopez J.R., Takeshima H., Ma J., Allen P.D., Eltit J.M. (2010). Impaired Orai1-mediated resting Ca2+ entry reduces the cytosolic [Ca2+] and sarcoplasmic reticulum Ca2+ loading in quiescent junctophilin 1 knock-out myotubes. J. Biol. Chem..

[49] Ahn M.K., Lee K.J., Cai C., Huang M., Cho C.H., Ma J., Lee E.H. (2016). Mitsugumin 53 regulates extracellular Ca(2+) entry and intracellular Ca(2+) release via Orai1 and RyR1 in skeletal muscle. Sci. Rep..

[50] Zhao X., Weisleder N., Thornton A., Oppong Y., Campbell R., Ma J., Brotto M. (2008). Compromised store-operated Ca2+ entry in aged skeletal muscle. Aging Cell.

[51] Darbellay B., Arnaudeau S., Ceroni D., Bader C.R., Konig S., Bernheim L. (2010). Human muscle economy myoblast differentiation and excitation-contraction coupling use the same molecular partners, STIM1 and STIM2. J. Biol. Chem..

[52] Horinouchi T., Higashi T., Higa T., Terada K., Mai Y., Aoyagi H., Hatate C., Nepal P., Horiguchi M., Harada T. (2012). Different binding property of STIM1 and its novel splice variant STIM1L to Orai1, TRPC3, and TRPC6 channels. Biochem. Biophys. Res. Commun..

[53] Antigny F., Koenig S., Bernheim L., Frieden M. (2013). During post-natal human myogenesis, normal myotube size requires TRPC1- and TRPC4-mediated Ca(2)(+) entry. J. Cell Sci..

[54] Darbellay B., Arnaudeau S., Konig S., Jousset H., Bader C., Demaurex N., Bernheim L. (2009). STIM1- and Orai1-dependent store-operated calcium entry regulates human myoblast differentiation. J. Biol. Chem..

[55] Li T., Finch E.A., Graham V., Zhang Z.S., Ding J.D., Burch J., Oh-hora M., Rosenberg P. (2012). STIM1-Ca(2+) signaling is required for the hypertrophic growth of skeletal muscle in mice. Mol. Cell Biol..

[56] Lacruz R.S., Feske S. (2015). Diseases caused by mutations in ORAI1 and STIM1. Ann. N. Y. Acad. Sci..

[57] Edwards J.N., Friedrich O., Cully T.R., von Wegner F., Murphy R.M., Launikonis B.S. (2010). Upregulation of store-operated Ca2+ entry in dystrophic mdx mouse muscle. Am. J. Physiol. Cell Physiol..

[58] Onopiuk M., Brutkowski W., Young C., Krasowska E., Rog J., Ritso M., Wojciechowska S., Arkle S., Zablocki K., Gorecki D.C. (2015). Store-operated calcium entry contributes to abnormal Ca(2)(+) signalling in dystrophic mdx mouse myoblasts. Arch. Biochem. Biophys..

[59] Choi J.H., Huang M., Hyun C., Oh M.R., Lee K.J., Cho C.H., Lee E.H. (2019). A muscular hypotonia-associated STIM1 mutant at R429 induces abnormalities in intracellular Ca(2+) movement and extracellular Ca(2+) entry in skeletal muscle. Sci. Rep..

[60] Lee K.J., Hyun C., Woo J.S., Park C.S., Kim D.H., Lee E.H. (2014). Stromal interaction molecule 1 (STIM1) regulates sarcoplasmic/endoplasmic reticulum Ca(2)(+)-ATPase 1a (SERCA1a) in skeletal muscle. Pflugers Arch..

[61] Sauc S., Frieden M. (2017). Neurological and Motor Disorders: TRPC in the Skeletal Muscle. Adv. Exp. Med. Biol..

[62] Oh M.R., Lee K.J., Huang M., Kim J.O., Kim D.H., Cho C.H., Lee E.H. (2017). STIM2 regulates both intracellular Ca(2+) distribution and Ca(2+) movement in skeletal myotubes. Sci. Rep..

[63] Phuong T.T.T., Kang T.M. (2015). Stromal interaction molecule 2 regulates C2C12 myoblast differentiation. Integr. Med. Res..

[64] Avila-Medina J., Mayoral-Gonzalez I., Dominguez-Rodriguez A., Gallardo-Castillo I., Ribas J., Ordonez A., Rosado J.A., Smani T. (2018). The Complex Role of Store Operated Calcium Entry Pathways and Related Proteins in the Function of Cardiac, Skeletal and Vascular Smooth Muscle Cells. Front. Physiol..

[65] Damann N., Voets T., Nilius B. (2008). TRPs in our senses. Curr. Biol..

[66] Minke B., Cook B. (2002). TRP channel proteins and signal transduction. Physiol. Rev..

[67] Montell C. (2005). The TRP superfamily of cation channels. Sci. STKE.

[68] Montell C. (2003). The venerable inveterate invertebrate TRP channels. Cell Calcium.

[69] Venkatachalam K., Montell C. (2007). TRP channels. Annu Rev. Biochem..

[70] Clapham D.E. (2007). Calcium signaling. Cell.

[71] Montell C., Birnbaumer L., Flockerzi V. (2002). The TRP channels, a remarkably functional family. Cell.

[72] Minke B. (2010). The history of the Drosophila TRP channel: The birth of a new channel superfamily. J. Neurogenet..

[73] Pedersen S.F., Owsianik G., Nilius B. (2005). TRP channels: An overview. Cell Calcium.

[74] Owsianik G., D’Hoedt D., Voets T., Nilius B. (2006). Structure-function relationship of the TRP channel superfamily. Rev. Physiol. Biochem. Pharmacol..

[75] Wissenbach U., Niemeyer B., Himmerkus N., Fixemer T., Bonkhoff H., Flockerzi V. (2004). TRPV6 and prostate cancer: Cancer growth beyond the prostate correlates with increased TRPV6 Ca2+ channel expression. Biochem. Biophys. Res. Commun..

[76] Abramowitz J., Birnbaumer L. (2009). Physiology and pathophysiology of canonical transient receptor potential channels. FASEB J..

[77] Vazquez G., Wedel B.J., Aziz O., Trebak M., Putney J.W. (2004). The mammalian TRPC cation channels. Biochim. Biophys. Acta.

[78] Schindl R., Romanin C. (2007). Assembly domains in TRP channels. Biochem. Soc. Trans..

[79] Zhu M.X. (2005). Multiple roles of calmodulin and other Ca(2+)-binding proteins in the functional regulation of TRP channels. Pflugers Arch..

[80] Singh B.B., Liu X., Tang J., Zhu M.X., Ambudkar I.S. (2002). Calmodulin regulates Ca(2+)-dependent feedback inhibition of store-operated Ca(2+) influx by interaction with a site in the C terminus of TrpC1. Mol. Cell.

[81] Beech D.J. (2013). Characteristics of transient receptor potential canonical calcium-permeable channels and their relevance to vascular physiology and disease. Circ. J..

[82] Odell A.F., Scott J.L., Van Helden D.F. (2005). Epidermal growth factor induces tyrosine phosphorylation, membrane insertion, and activation of transient receptor potential channel 4. J. Biol. Chem..

[83] Lockwich T.P., Liu X., Singh B.B., Jadlowiec J., Weiland S., Ambudkar I.S. (2000). Assembly of Trp1 in a signaling complex associated with caveolin-scaffolding lipid raft domains. J. Biol. Chem..

[84] Hofmann T., Obukhov A.G., Schaefer M., Harteneck C., Gudermann T., Schultz G. (1999). Direct activation of human TRPC6 and TRPC3 channels by diacylglycerol. Nature.

[85] Graham S., Ding M., Ding Y., Sours-Brothers S., Luchowski R., Gryczynski Z., Yorio T., Ma H., Ma R. (2010). Canonical transient receptor potential 6 (TRPC6), a redox-regulated cation channel. J. Biol. Chem..

[86] Bon R.S., Beech D.J. (2013). In pursuit of small molecule chemistry for calcium-permeable non-selective TRPC channels -- mirage or pot of gold?. Br. J. Pharmacol..

[87] Harteneck C., Plant T.D., Schultz G. (2000). From worm to man: Three subfamilies of TRP channels. Trends Neurosci..

[88] Singh B.B., Lockwich T.P., Bandyopadhyay B.C., Liu X., Bollimuntha S., Brazer S.C., Combs C., Das S., Leenders A.G., Sheng Z.H. (2004). VAMP2-dependent exocytosis regulates plasma membrane insertion of TRPC3 channels and contributes to agonist-stimulated Ca2+ influx. Mol. Cell.

[89] Van Rossum D.B., Patterson R.L., Sharma S., Barrow R.K., Kornberg M., Gill D.L., Snyder S.H. (2005). Phospholipase Cgamma1 controls surface expression of TRPC3 through an intermolecular PH domain. Nature.

[90] Bezzerides V.J., Ramsey I.S., Kotecha S., Greka A., Clapham D.E. (2004). Rapid vesicular translocation and insertion of TRP channels. Nat. Cell Biol..

[91] Dietrich A., Mederos y Schnitzler M., Emmel J., Kalwa H., Hofmann T., Gudermann T. (2003). N-linked protein glycosylation is a major determinant for basal TRPC3 and TRPC6 channel activity. J. Biol. Chem..

[92] Sukumar P., Sedo A., Li J., Wilson L.A., O’Regan D., Lippiat J.D., Porter K.E., Kearney M.T., Ainscough J.F., Beech D.J. (2012). Constitutively active TRPC channels of adipocytes confer a mechanism for sensing dietary fatty acids and regulating adiponectin. Circ. Res..

[93] Philipp S., Cavalie A., Freichel M., Wissenbach U., Zimmer S., Trost C., Marquart A., Murakami M., Flockerzi V. (1996). A mammalian capacitative calcium entry channel homologous to Drosophila TRP and TRPL. EMBO J..

[94] Warnat J., Philipp S., Zimmer S., Flockerzi V., Cavalie A. (1999). Phenotype of a recombinant store-operated channel: Highly selective permeation of Ca2+. J. Physiol..

[95] Freichel M., Suh S.H., Pfeifer A., Schweig U., Trost C., Weissgerber P., Biel M., Philipp S., Freise D., Droogmans G. (2001). Lack of an endothelial store-operated Ca2+ current impairs agonist-dependent vasorelaxation in TRP4-/- mice. Nat. Cell Biol..

[96] Vazquez G., Lievremont J.P., St J.B.G., Putney J.W. (2001). Human Trp3 forms both inositol trisphosphate receptor-dependent and receptor-independent store-operated cation channels in DT40 avian B lymphocytes. Proc. Natl. Acad. Sci. USA.

[97] Zitt C., Zobel A., Obukhov A.G., Harteneck C., Kalkbrenner F., Luckhoff A., Schultz G. (1996). Cloning and functional expression of a human Ca2+-permeable cation channel activated by calcium store depletion. Neuron.

[98] Franco A., Lansman J.B. (1990). Calcium entry through stretch-inactivated ion channels in mdx myotubes. Nature.

[99] Vandebrouck C., Duport G., Cognard C., Raymond G. (2001). Cationic channels in normal and dystrophic human myotubes. Neuromuscul. Disord..

[100] Tutdibi O., Brinkmeier H., Rudel R., Fohr K.J. (1999). Increased calcium entry into dystrophin-deficient muscle fibres of MDX and ADR-MDX mice is reduced by ion channel blockers. J. Physiol..

[101] Maroto R., Raso A., Wood T.G., Kurosky A., Martinac B., Hamill O.P. (2005). TRPC1 forms the stretch-activated cation channel in vertebrate cells. Nat. Cell Biol..

[102] Wilson C., Dryer S.E. (2014). A mutation in TRPC6 channels abolishes their activation by hypoosmotic stretch but does not affect activation by diacylglycerol or G protein signaling cascades. Am. J. Physiol. Renal Physiol..

[103] Fels B., Nielsen N., Schwab A. (2016). Role of TRPC1 channels in pressure-mediated activation of murine pancreatic stellate cells. Eur. Biophys. J..

[104] Li N., He Y., Yang G., Yu Q., Li M. (2019). Role of TRPC1 channels in pressure-mediated activation of airway remodeling. Respir Res..

[105] Molnar T., Yarishkin O., Iuso A., Barabas P., Jones B., Marc R.E., Phuong T.T., Krizaj D. (2016). Store-Operated Calcium Entry in Muller Glia Is Controlled by Synergistic Activation of TRPC and Orai Channels. J. Neurosci..

[106] Staaf S., Maxvall I., Lind U., Husmark J., Mattsson J.P., Ernfors P., Pierrou S. (2009). Down regulation of TRPC1 by shRNA reduces mechanosensitivity in mouse dorsal root ganglion neurons in vitro. Neurosci. Lett..

[107] Formigli L., Sassoli C., Squecco R., Bini F., Martinesi M., Chellini F., Luciani G., Sbrana F., Zecchi-Orlandini S., Francini F. (2009). Regulation of transient receptor potential canonical channel 1 (TRPC1) by sphingosine 1-phosphate in C2C12 myoblasts and its relevance for a role of mechanotransduction in skeletal muscle differentiation. J. Cell Sci..

[108] Gottlieb P., Folgering J., Maroto R., Raso A., Wood T.G., Kurosky A., Bowman C., Bichet D., Patel A., Sachs F. (2008). Revisiting TRPC1 and TRPC6 mechanosensitivity. Pflugers Arch..

[109] Mederos y Schnitzler M., Storch U., Meibers S., Nurwakagari P., Breit A., Essin K., Gollasch M., Gudermann T. (2008). Gq-coupled receptors as mechanosensors mediating myogenic vasoconstriction. EMBO J..

[110] Kruger J., Kunert-Keil C., Bisping F., Brinkmeier H. (2008). Transient receptor potential cation channels in normal and dystrophic mdx muscle. Neuromuscul. Disord..

[111] Brinkmeier H. (2011). TRP channels in skeletal muscle: Gene expression, function and implications for disease. Adv. Exp. Med. Biol..

[112] Gailly P. (2012). TRP channels in normal and dystrophic skeletal muscle. Curr. Opin. Pharmacol..

[113] Zanou N., Shapovalov G., Louis M., Tajeddine N., Gallo C., Van Schoor M., Anguish I., Cao M.L., Schakman O., Dietrich A. (2010). Role of TRPC1 channel in skeletal muscle function. Am. J. Physiol. Cell Physiol..

[114] Ahmad A.A., Streiff M., Hunter C., Hu Q., Sachse F.B. (2017). Physiological and pathophysiological role of transient receptor potential canonical channels in cardiac myocytes. Prog. Biophys. Mol. Biol..

[115] Avila-Medina J., Mayoral-Gonzalez I., Galeano-Otero I., Redondo P.C., Rosado J.A., Smani T. (2020). Pathophysiological Significance of Store-Operated Calcium Entry in Cardiovascular and Skeletal Muscle Disorders and Angiogenesis. Adv. Exp. Med. Biol..

[116] Jansen K.M., Pavlath G.K. (2008). Molecular control of mammalian myoblast fusion. Methods Mol. Biol..

[117] Schollmeyer J.E. (1986). Role of Ca2+ and Ca2+-activated protease in myoblast fusion. Exp. Cell Res..

[118] Wakelam M.J. (1985). The fusion of myoblasts. Biochem. J..

[119] Louis M., Zanou N., Van Schoor M., Gailly P. (2008). TRPC1 regulates skeletal myoblast migration and differentiation. J. Cell Sci..

[120] Olah T., Fodor J., Ruzsnavszky O., Vincze J., Berbey C., Allard B., Csernoch L. (2011). Overexpression of transient receptor potential canonical type 1 (TRPC1) alters both store operated calcium entry and depolarization-evoked calcium signals in C2C12 cells. Cell Calcium.

[121] Ma R., Rundle D., Jacks J., Koch M., Downs T., Tsiokas L. (2003). Inhibitor of myogenic family, a novel suppressor of store-operated currents through an interaction with TRPC1. J. Biol. Chem..

[122] Xia L., Cheung K.K., Yeung S.S., Yeung E.W. (2016). The involvement of transient receptor potential canonical type 1 in skeletal muscle regrowth after unloading-induced atrophy. J. Physiol..

[123] Zhang B.T., Yeung S.S., Cheung K.K., Chai Z.Y., Yeung E.W. (2014). Adaptive responses of TRPC1 and TRPC3 during skeletal muscle atrophy and regrowth. Muscle Nerve.

[124] Zanou N., Schakman O., Louis P., Ruegg U.T., Dietrich A., Birnbaumer L., Gailly P. (2012). Trpc1 ion channel modulates phosphatidylinositol 3-kinase/Akt pathway during myoblast differentiation and muscle regeneration. J. Biol. Chem..

[125] Liu Y., Schneider M.F. (2014). FGF2 activates TRPC and Ca(2+) signaling leading to satellite cell activation. Front. Physiol..

[126] Imaoka Y., Kawai M., Mori F., Miyata H. (2015). Effect of eccentric contraction on satellite cell activation in human vastus lateralis muscle. J. Physiol Sci.

[127] Woo J.S., Kim D.H., Allen P.D., Lee E.H. (2008). TRPC3-interacting triadic proteins in skeletal muscle. Biochem. J..

[128] Sabourin J., Lamiche C., Vandebrouck A., Magaud C., Rivet J., Cognard C., Bourmeyster N., Constantin B. (2009). Regulation of TRPC1 and TRPC4 cation channels requires an alpha1-syntrophin-dependent complex in skeletal mouse myotubes. J. Biol. Chem..

[129] Woo J.S., Cho C.H., Kim D.H., Lee E.H. (2010). TRPC3 cation channel plays an important role in proliferation and differentiation of skeletal muscle myoblasts. Exp. Mol. Med..

[130] Woo J.S., Lee K.J., Huang M., Cho C.H., Lee E.H. (2014). Heteromeric TRPC3 with TRPC1 formed via its ankyrin repeats regulates the resting cytosolic Ca2+ levels in skeletal muscle. Biochem. Biophys. Res. Commun..

[131] Rosenberg P., Hawkins A., Stiber J., Shelton J.M., Hutcheson K., Bassel-Duby R., Shin D.M., Yan Z., Williams R.S. (2004). TRPC3 channels confer cellular memory of recent neuromuscular activity. Proc. Natl. Acad. Sci. USA.

[132] Hogarth M.W., Houweling P.J., Thomas K.C., Gordish-Dressman H., Bello L., Cooperative International Neuromuscular Research G., Pegoraro E., Hoffman E.P., Head S.I., North K.N. (2017). Evidence for ACTN3 as a genetic modifier of Duchenne muscular dystrophy. Nat. Commun..

[133] Lanner J.T., Bruton J.D., Assefaw-Redda Y., Andronache Z., Zhang S.J., Severa D., Zhang Z.B., Melzer W., Zhang S.L., Katz A. (2009). Knockdown of TRPC3 with siRNA coupled to carbon nanotubes results in decreased insulin-mediated glucose uptake in adult skeletal muscle cells. FASEB J..

[134] Lee E.H., Cherednichenko G., Pessah I.N., Allen P.D. (2006). Functional coupling between TRPC3 and RyR1 regulates the expressions of key triadic proteins. J. Biol. Chem..

[135] Sampieri A., Diaz-Munoz M., Antaramian A., Vaca L. (2005). The foot structure from the type 1 ryanodine receptor is required for functional coupling to store-operated channels. J. Biol. Chem..

[136] Dietrich A., Gudermann T. (2007). Trpc6. Handb Exp. Pharmacol..

[137] Spassova M.A., Hewavitharana T., Xu W., Soboloff J., Gill D.L. (2006). A common mechanism underlies stretch activation and receptor activation of TRPC6 channels. Proc. Natl. Acad. Sci. USA.

[138] Pedersen S.F., Nilius B. (2007). Transient receptor potential channels in mechanosensing and cell volume regulation. Methods Enzymol..

[139] Winn M.P., Conlon P.J., Lynn K.L., Farrington M.K., Creazzo T., Hawkins A.F., Daskalakis N., Kwan S.Y., Ebersviller S., Burchette J.L. (2005). A mutation in the TRPC6 cation channel causes familial focal segmental glomerulosclerosis. Science.

[140] Reiser J., Polu K.R., Moller C.C., Kenlan P., Altintas M.M., Wei C., Faul C., Herbert S., Villegas I., Avila-Casado C. (2005). TRPC6 is a glomerular slit diaphragm-associated channel required for normal renal function. Nat. Genet..

[141] Yu Y., Keller S.H., Remillard C.V., Safrina O., Nicholson A., Zhang S.L., Jiang W., Vangala N., Landsberg J.W., Wang J.Y. (2009). A functional single-nucleotide polymorphism in the TRPC6 gene promoter associated with idiopathic pulmonary arterial hypertension. Circulation.

[142] Emery A.E. (2002). The muscular dystrophies. Lancet.

[143] Blake D.J., Weir A., Newey S.E., Davies K.E. (2002). Function and genetics of dystrophin and dystrophin-related proteins in muscle. Physiol. Rev..

[144] Campbell K.P. (1995). Three muscular dystrophies: Loss of cytoskeleton-extracellular matrix linkage. Cell.

[145] Ervasti J.M., Sonnemann K.J. (2008). Biology of the striated muscle dystrophin-glycoprotein complex. Int. Rev. Cytol..

[146] Hoffman E.P., Brown R.H., Kunkel L.M. (1987). Dystrophin: The protein product of the Duchenne muscular dystrophy locus. Cell.

[147] Moens P., Baatsen P.H., Marechal G. (1993). Increased susceptibility of EDL muscles from mdx mice to damage induced by contractions with stretch. J. Muscle Res. Cell Motil..

[148] Allard B. (2006). Sarcolemmal ion channels in dystrophin-deficient skeletal muscle fibres. J. Muscle Res. Cell Motil..

[149] Ruegg U.T., Gillis J.M. (1999). Calcium homeostasis in dystrophic muscle. Trends Pharmacol. Sci..

[150] Gailly P. (2002). New aspects of calcium signaling in skeletal muscle cells: Implications in Duchenne muscular dystrophy. Biochim. Biophys. Acta.

[151] Gillis J.M. (1999). Understanding dystrophinopathies: An inventory of the structural and functional consequences of the absence of dystrophin in muscles of the mdx mouse. J. Muscle Res. Cell Motil..

[152] Lansman J.B., Franco A. (1991). What does dystrophin do in normal muscle?. J. Muscle Res. Cell Motil..

[153] Petrof B.J., Shrager J.B., Stedman H.H., Kelly A.M., Sweeney H.L. (1993). Dystrophin protects the sarcolemma from stresses developed during muscle contraction. Proc. Natl. Acad. Sci. USA.

[154] Proske U., Morgan D.L. (2001). Muscle damage from eccentric exercise: Mechanism, mechanical signs, adaptation and clinical applications. J. Physiol..

[155] Oberc M.A., Engel W.K. (1977). Ultrastructural localization of calcium in normal and abnormal skeletal muscle. Lab. Invest..

[156] Robert V., Massimino M.L., Tosello V., Marsault R., Cantini M., Sorrentino V., Pozzan T. (2001). Alteration in calcium handling at the subcellular level in mdx myotubes. J. Biol. Chem..

[157] Mallouk N., Jacquemond V., Allard B. (2000). Elevated subsarcolemmal Ca2+ in mdx mouse skeletal muscle fibers detected with Ca2+-activated K+ channels. Proc. Natl. Acad. Sci. USA.

[158] Mongini T., Ghigo D., Doriguzzi C., Bussolino F., Pescarmona G., Pollo B., Schiffer D., Bosia A. (1988). Free cytoplasmic Ca++ at rest and after cholinergic stimulus is increased in cultured muscle cells from Duchenne muscular dystrophy patients. Neurology.

[159] Turner P.R., Westwood T., Regen C.M., Steinhardt R.A. (1988). Increased protein degradation results from elevated free calcium levels found in muscle from mdx mice. Nature.

[160] Williams D.A., Head S.I., Bakker A.J., Stephenson D.G. (1990). Resting calcium concentrations in isolated skeletal muscle fibres of dystrophic mice. J. Physiol..

[161] Sandri M., El Meslemani A.H., Sandri C., Schjerling P., Vissing K., Andersen J.L., Rossini K., Carraro U., Angelini C. (2001). Caspase 3 expression correlates with skeletal muscle apoptosis in Duchenne and facioscapulo human muscular dystrophy. A potential target for pharmacological treatment?. J. Neuropathol. Exp. Neurol..

[162] Mikhailov V.M., Kropotov A.V., Zelenin A.V., Krutilina R.I., Kolesnikov V.A., Zelenina I.A., Baranov A.N., Shtein G.I., Ostapenko O.V., Tomilin N.V. (2002). The BCL-xL and ACR-1 genes promote differentiation and reduce apoptosis in muscle fibers of mdx mice. Genetika.

[163] Sandri M., Carraro U. (1999). Apoptosis of skeletal muscles during development and disease. Int. J. Biochem. Cell Biol..

[164] Collins C.A., Morgan J.E. (2003). Duchenne’s muscular dystrophy: Animal models used to investigate pathogenesis and develop therapeutic strategies. Int. J. Exp. Pathol..

[165] Imbert N., Vandebrouck C., Constantin B., Duport G., Guillou C., Cognard C., Raymond G. (1996). Hypoosmotic shocks induce elevation of resting calcium level in Duchenne muscular dystrophy myotubes contracting in vitro. Neuromuscul. Disord..

[166] Gailly P., Boland B., Himpens B., Casteels R., Gillis J.M. (1993). Critical evaluation of cytosolic calcium determination in resting muscle fibres from normal and dystrophic (mdx) mice. Cell Calcium.

[167] Mallouk N., Allard B. (2000). Stretch-induced activation of Ca(2+)-activated K(+) channels in mouse skeletal muscle fibers. Am. J. Physiol. Cell Physiol..

[168] Gailly P., De Backer F., Van Schoor M., Gillis J.M. (2007). In situ measurements of calpain activity in isolated muscle fibres from normal and dystrophin-lacking mdx mice. J. Physiol..

[169] Vandebrouck A., Ducret T., Basset O., Sebille S., Raymond G., Ruegg U., Gailly P., Cognard C., Constantin B. (2006). Regulation of store-operated calcium entries and mitochondrial uptake by minidystrophin expression in cultured myotubes. FASEB J..

[170] Gervasio O.L., Whitehead N.P., Yeung E.W., Phillips W.D., Allen D.G. (2008). TRPC1 binds to caveolin-3 and is regulated by Src kinase - role in Duchenne muscular dystrophy. J. Cell Sci..

[171] Allen D.G., Whitehead N.P. (2011). Duchenne muscular dystrophy--what causes the increased membrane permeability in skeletal muscle?. Int. J. Biochem. Cell Biol..

[172] Allen D.G., Whitehead N.P., Yeung E.W. (2005). Mechanisms of stretch-induced muscle damage in normal and dystrophic muscle: Role of ionic changes. J. Physiol..

[173] Gailly P., Hermans E., Octave J.N., Gillis J.M. (1993). Specific increase of genetic expression of parvalbumin in fast skeletal muscles of mdx mice. FEBS Lett.

[174] Schiavone M., Zulian A., Menazza S., Petronilli V., Argenton F., Merlini L., Sabatelli P., Bernardi P. (2017). Alisporivir rescues defective mitochondrial respiration in Duchenne muscular dystrophy. Pharmacol. Res..

[175] Alderton J.M., Steinhardt R.A. (2000). Calcium influx through calcium leak channels is responsible for the elevated levels of calcium-dependent proteolysis in dystrophic myotubes. J. Biol. Chem..

[176] Fong P.Y., Turner P.R., Denetclaw W.F., Steinhardt R.A. (1990). Increased activity of calcium leak channels in myotubes of Duchenne human and mdx mouse origin. Science.

[177] Hopf F.W., Reddy P., Hong J., Steinhardt R.A. (1996). A capacitative calcium current in cultured skeletal muscle cells is mediated by the calcium-specific leak channel and inhibited by dihydropyridine compounds. J. Biol. Chem..

[178] Wang X., Weisleder N., Collet C., Zhou J., Chu Y., Hirata Y., Zhao X., Pan Z., Brotto M., Cheng H. (2005). Uncontrolled calcium sparks act as a dystrophic signal for mammalian skeletal muscle. Nat. Cell Biol..

[179] Franco-Obregon A., Lansman J.B. (1994). Mechanosensitive ion channels in skeletal muscle from normal and dystrophic mice. J. Physiol..

[180] Rolland J.F., De Luca A., Burdi R., Andreetta F., Confalonieri P., Conte Camerino D. (2006). Overactivity of exercise-sensitive cation channels and their impaired modulation by IGF-1 in mdx native muscle fibers: Beneficial effect of pentoxifylline. Neurobiol. Dis..

[181] Watkins S.C., Cullen M.J. (1987). A qualitative and quantitative study of the ultrastructure of regenerating muscle fibres in Duchenne muscular dystrophy and polymyositis. J. Neurol. Sci..

[182] Kuznetsov A.V., Winkler K., Wiedemann F.R., von Bossanyi P., Dietzmann K., Kunz W.S. (1998). Impaired mitochondrial oxidative phosphorylation in skeletal muscle of the dystrophin-deficient mdx mouse. Mol. Cell Biochem..

[183] Zulian A., Schiavone M., Giorgio V., Bernardi P. (2016). Forty years later: Mitochondria as therapeutic targets in muscle diseases. Pharmacol. Res..

[184] Millay D.P., Goonasekera S.A., Sargent M.A., Maillet M., Aronow B.J., Molkentin J.D. (2009). Calcium influx is sufficient to induce muscular dystrophy through a TRPC-dependent mechanism. Proc. Natl. Acad. Sci. USA.

[185] Whitehead N.P., Yeung E.W., Froehner S.C., Allen D.G. (2010). Skeletal muscle NADPH oxidase is increased and triggers stretch-induced damage in the mdx mouse. PLoS ONE.

[186] Vandebrouck A., Sabourin J., Rivet J., Balghi H., Sebille S., Kitzis A., Raymond G., Cognard C., Bourmeyster N., Constantin B. (2007). Regulation of capacitative calcium entries by alpha1-syntrophin: Association of TRPC1 with dystrophin complex and the PDZ domain of alpha1-syntrophin. FASEB J..

[187] Adams M.E., Butler M.H., Dwyer T.M., Peters M.F., Murnane A.A., Froehner S.C. (1993). Two forms of mouse syntrophin, a 58 kd dystrophin-associated protein, differ in primary structure and tissue distribution. Neuron.

[188] Boittin F.X., Petermann O., Hirn C., Mittaud P., Dorchies O.M., Roulet E., Ruegg U.T. (2006). Ca2+-independent phospholipase A2 enhances store-operated Ca2+ entry in dystrophic skeletal muscle fibers. J. Cell Sci..

[189] Vaghy P.L., Fang J., Wu W., Vaghy L.P. (1998). Increased caveolin-3 levels in mdx mouse muscles. FEBS Lett..

[190] Venema V.J., Ju H., Zou R., Venema R.C. (1997). Interaction of neuronal nitric-oxide synthase with caveolin-3 in skeletal muscle. Identification of a novel caveolin scaffolding/inhibitory domain. J. Biol Chem.

[191] Shibuya S., Wakayama Y., Inoue M., Oniki H., Kominami E. (2002). Changes in the distribution and density of caveolin 3 molecules at the plasma membrane of mdx mouse skeletal muscles: A fracture-label electron microscopic study. Neurosci. Lett..

[192] Galbiati F., Razani B., Lisanti M.P. (2001). Caveolae and caveolin-3 in muscular dystrophy. Trends Mol. Med..

[193] Allen D.G., Gervasio O.L., Yeung E.W., Whitehead N.P. (2010). Calcium and the damage pathways in muscular dystrophy. Can. J. Physiol. Pharmacol..

[194] Sotgia F., Lee J.K., Das K., Bedford M., Petrucci T.C., Macioce P., Sargiacomo M., Bricarelli F.D., Minetti C., Sudol M. (2000). Caveolin-3 directly interacts with the C-terminal tail of beta -dystroglycan. Identification of a central WW-like domain within caveolin family members. J. Biol. Chem..

[195] Stiber J.A., Tabatabaei N., Hawkins A.F., Hawke T., Worley P.F., Williams R.S., Rosenberg P. (2005). Homer modulates NFAT-dependent signaling during muscle differentiation. Dev. Biol..

[196] Stiber J.A., Zhang Z.S., Burch J., Eu J.P., Zhang S., Truskey G.A., Seth M., Yamaguchi N., Meissner G., Shah R. (2008). Mice lacking Homer 1 exhibit a skeletal myopathy characterized by abnormal transient receptor potential channel activity. Mol. Cell Biol..

[197] Bidou L., Allamand V., Rousset J.P., Namy O. (2012). Sense from nonsense: Therapies for premature stop codon diseases. Trends Mol. Med..

[198] Wagner K.R., Hamed S., Hadley D.W., Gropman A.L., Burstein A.H., Escolar D.M., Hoffman E.P., Fischbeck K.H. (2001). Gentamicin treatment of Duchenne and Becker muscular dystrophy due to nonsense mutations. Ann. Neurol..

[199] Finkel R.S. (2010). Read-through strategies for suppression of nonsense mutations in Duchenne/ Becker muscular dystrophy: Aminoglycosides and ataluren (PTC124). J. Child. Neurol..

[200] Marchand E., Constantin B., Balghi H., Claudepierre M.C., Cantereau A., Magaud C., Mouzou A., Raymond G., Braun S., Cognard C. (2004). Improvement of calcium handling and changes in calcium-release properties after mini- or full-length dystrophin forced expression in cultured skeletal myotubes. Exp. Cell Res..

[201] Mendell J.R., Campbell K., Rodino-Klapac L., Sahenk Z., Shilling C., Lewis S., Bowles D., Gray S., Li C., Galloway G. (2010). Dystrophin immunity in Duchenne’s muscular dystrophy. N. Engl. J. Med..

